# Some Aspects of Intestinal Obstruction

**Published:** 1908-08-08

**Authors:** 


### SOME ASPECTS OF INTESTINAL OBSTRUCTION.

### GENERAL TREATMENT OF A CASE IN WHICH OPERATION HAS BEEN REFUSED; AND THE CHOICE OF OPERATIONS.

In cases of chronic obstruction due to growth in the colon, where operative interference has been refused the feeding of the patient is a matter of the utmost importance. The lumen of the intestine is only partially occluded, so that fluid matter of soft consistence can pass through the obstruction, but any large solid particles will plug the stenosed part and at once produce acute symptoms. If the patient exercises extreme care in the selection of his food he will enjoy immunity from severe trouble until such time as the condition of the stricture will no longer allow the passage of even well-digested matter, and in this way he may hold his ground for some considerable time.

He should take an increased number of small meals rather than a few large ones. The food should be such as can be easily digested, and *will leave the minimum of residual debris*. It may be peptonised or otherwise artificially digested, in order to put the least possible strain on the digestive organs. Meat usually causes pain, and should be replaced by chicken and fish, which, as the stenosis increases, has to be minced.

Next only fluid preparations can be taken, and the patient is forced to live on peptonised milk, beef-tea, jelly, and meat extracts. In due course all food gives distress, and the patient becomes wasted and emaciated from lack of nutrition. At this point it becomes essential to feed him with nutrient enemata. One which will be found very satisfactory is composed as follows: Milk ℥ij, beef tea ℥ij, and an egg, peptonised, with or without a little brandy. The rectum must be cleared daily with a wash-out enema. The stage when rectal feeding is reached must be regarded as the beginning of the end, and the patient rarely lives more than a few weeks from this time.

The treatment of the pain caused by the peristaltic action of the intestine, which is intermittent at first, but almost constant, and very severe towards the end, is a matter of some difficulty. The only drug on which any real reliance can be placed is morphia; but morphia adds to the trouble because it stills the intestinal movements, and encourages an accumulation of bowel contents above the obstruction, while it discourages the emptying of the bowel below. It cannot, however, be withheld for long, lest the pain, which harries the patient all day and keeps him awake all night, proves fatal.

It is most important to see that the bowels are kept open. Aperients given by the mouth tend to induce peristalsis and increase the pain; and they are not without danger, for an aperient has before now been the determining factor in precipitating an attack of acute obstruction on top of a chronic one. Calomel and castor oil are the best, if aperients must be used. But the safest way of keeping the bowels open is by the use of enemata-operative treatment.

### Operative Treatment.

The choice of treatment in a case of chronic intestinal obstruction is entirely different according to whether the obstruction is complete or not. When it is still incomplete the ideal method is to excise the stricture whether it be simple or malignant, and to do an end-to-end anastomosis, while the health of the patient is still fairly good. If the existence of a tumour can be made out there must be no delay in opening the abdomen, since the earlier the diagnosis is made the greater will be the chance of removing the growth.

The abdomen is best opened just to one side of the middle line through the right or left rectus muscle. This incision is preferable to a median one because firmer union ensues, and if it is necessary to enlarge the incision upwards the umbilicus is not in the way. If a tumour can be definitely palpated on the right or left iliac fossa, the incision may be made near the outer edge of the corresponding rectus muscle. When the abdomen is opened search must be made for the area of stenosis. In the first place any tumour which may have been palpated through the parietes should be felt for. As often as not none will be found in the place where it had been expected; although before the operation and even under the anæsthetic it may have felt extremely definite.

In such an event the surgeon should proceed next to palpate the cæcum. If this is collapsed it may be taken for granted that the obstruction is in the small intestine, and conversely if it is distended the seat of the trouble is somewhere in the large gut. It is next necessary to pass the intestine bit by bit through the hands, carefully replacing each portion as it is examined into the peritoneal cavity. This sounds an easy thing to do, but in practice it is much more often the reverse, and anyone who has watched these operations cannot but appreciate the difficulty which is apt to arise in actually locating the disease. In extreme cases it may be necessary to bring out the intestines in warm towels and search for the obstruction in the open.

When it has been found the question arises: What operative measure gives the patient the best chance? If the growth is apparently innocent or if it is a carcinoma where neither the mesenteric glands nor the liver are yet affected, it should be completely excised. But a much more ambiguous answer must be given if the question is asked should excision be undertaken when the growth is quite movable, but the mesenteric glands are involved? Cheyne and Burghard reply in the affirmative, and give two reasons for their opinion. The patient, if nothing is done, will eventually suffer from complete obstruction, and have to undergo an emergency operation under far less advantageous circumstances, and they assert that secondary growths in this neighbourhood do not tend to grow rapidly. They make the reservation that their remarks do not apply to growths in fixed portions of the intestine, for they regard excision in such a situation with subsequent end-to-end anastomosis as a most difficult and dangerous operation.

